# A people‐centred perspective on climate change, environmental stress, and livelihood resilience in Bangladesh

**DOI:** 10.1007/s11625-016-0379-z

**Published:** 2016-06-11

**Authors:** Sonja Ayeb-Karlsson, Kees van der Geest, Istiakh Ahmed, Saleemul Huq, Koko Warner

**Affiliations:** 1 United Nations University Institute for Environment and Human Security (UNU-EHS), Bonn, Germany; 2 University of Sussex, Brighton, UK; 3 International Centre for Climate Change and Development (ICCCAD), Dhaka, Bangladesh

**Keywords:** Livelihood resilience, Climate change, Adaptation, Natural disasters, People-centred research

## Abstract

The Ganges–Brahmaputra delta enables Bangladesh to sustain a dense population, but it also exposes people to natural hazards. This article presents findings from the Gibika project, which researches livelihood resilience in seven study sites across Bangladesh. This study aims to understand how people in the study sites build resilience against environmental stresses, such as cyclones, floods, riverbank erosion, and drought, and in what ways their strategies sometimes fail. The article applies a new methodology for studying people’s decision making in risk-prone environments: the personal Livelihood History interviews (*N* = 28). The findings show how environmental stress, shocks, and disturbances affect people’s livelihood resilience and why adaptation measures can be unsuccessful. Floods, riverbank erosion, and droughts cause damage to agricultural lands, crops, houses, and properties. People manage to adapt by modifying their agricultural practices, switching to alternative livelihoods, or using migration as an adaptive strategy. In the coastal study sites, cyclones are a severe hazard. The study reveals that when a cyclone approaches, people sometimes choose not to evacuate: they put their lives at risk to protect their livelihoods and properties. Future policy and adaptation planning must use lessons learned from people currently facing environmental stress and shocks.

## Introduction

Nature has been kind to Bangladesh by positioning the country in the world’s largest delta. Out of the world’s 500 million people living in deltas, 100 million live in the Ganges–Brahmaputra delta in Bangladesh and India. Jamuna, Meghna, and other rivers criss-cross their way through the country before flowing out into the Bay of Bengal. Historically, the delta enables the country to sustain a dense population, but the delta location also exposes people to natural hazards. Cyclones, riverbank erosion, sea-level rise, land loss, and drought are all typical stressors in delta areas (Ahmed et al. 2012; Pouliotte et al. 2009; Lewis 2011).

This article provides insights from study sites spread across Bangladesh; Barisal, Khulna, Rajshahi, and Dhaka division.

Scientific evidence, synthesized in subsequent IPCC reports, shows that low-income countries are the most severely affected by climatic stress (Khan and Nahar 2014; Schneider et al. 2007; van der Geest and Warner 2015; IPCC WG2 AR5 2014). The soil, water, and land that people in climate change-affected areas depend on for their livelihoods are at risk of losing their ability to support humans and their societies (Baudoin et al. 2016; Costanza et al. 1997; Zommers et al. 2014). Poverty and food insecurity exacerbate vulnerability to natural hazards. Communities in poor countries are, therefore, hit harder by the effects of natural hazards and climate change (Basher 2006; Blaikie et al. 2014; Cannon 2000). Bangladesh ranked number 142 out of 188 countries on the 2014 Human Development Index, with a life expectancy of 71.6 years, 5.1 years of schooling, and a Gross National Income of $3191 PPP per capita (UNDP HDR 2015). According to the 2015 World Risk Index, the country is the sixth most at risk of natural disasters worldwide (Garschagen et al. 2015).

Livelihood resilience is a central concept in this article. In ecology, resilience is usually seen as a system’s ability to absorb and recover from disturbance (Folke et al. 2004; Gall 2013). The resilience process depends on a number of factors, such as previous condition, the time between disturbances, and their severity (Cutter et al. 2008). This study takes a people-centred perspective on resilience^1^ that emphasises not only the ability to absorb shocks and recover, but also livelihood improvement, despite disturbances. The definition of adaptation^2^ used in this article is in line with the one commonly referred to in the climate change field, also by the Intergovernmental Panel on Climate Change (IPCC). Adaptation must consider climate change, but it may not be to climate change alone; the informants in this study describe responding practices to environmental stress in Bangladesh. As the adaptation definition includes short-term coping as well as long-term transformations, they are also considered as adaptation strategies. In addition, this article defines disasters^3^ as natural hazards affecting vulnerable people (Blaikie et al. 2014; Cannon 2000; Cutter 2006; Cannon and Müller-Mahn 2010).

The research aim is to understand how people in the study sites can (and cannot) build resilience against a wide range of environmental stressors, in what ways their adaptation strategies sometimes fail and how we can learn from their lessons. A deeper understanding of people’s behaviour, when facing environmental stress, can provide valuable insights on people’s coping and adaptation constraints (Callahan and Elliott 1996; Patt and Schröter 2008; Patt and Schrag 2003). Qualitative research, involving a selection of 28 individuals, has been carried out through the personal Livelihood History interviews to enhance such behavioural understanding. The article material includes face-to-face interviews as well as textual analysis of the transcripts. There is currently a lack of people-centred research within the area of environmental science, climate change, and adaptation (Adger 2010; Oliver-Smith 1996). This journal article, therefore, is one of few that presents such a qualitative, people-centred perspective on climate change, environmental stress, and livelihood resilience in Bangladesh. In addition, it introduces and applies a new method for studying how people’s livelihoods and adaptation strategies evolve over time: The Livelihood History methodology.

## Research questions

By studying seven sites in Bangladesh, this research aims to advance the scientific understanding of how people either adapt or fail to adapt to environmental stress and shocks. This article tries to answer the following research questions:

1. How does environmental stress and climate change influence peoples’ livelihood resilience in Bangladesh?
2. How do people adapt to absorb and recover from environmental stress, shocks, and climate change impacts?
3. In what ways do these adaptation strategies strengthen people’s livelihood resilience, and in what ways do they fail?
4. How can insights on people’s adaptation strategies and constraints support future policy and adaptation planning?

The methodology selected to answer the research questions, personal Livelihood History interviews, provides a detailed analysis of people’s behaviour and responses to environmental stress and shocks. This method enables a deeper understanding of how and why people’s adaptation strategies fail. Policy makers depend on such insights while creating more sustainable climate change adaptation policies (Brown and Westaway 2011; Grothmann and Patt 2005; Adger 2010; Tompkins and Adger 2004). An objective of this article is, therefore, to show the added value of a people-centred approach to livelihood resilience. If one wishes to help increase the resilience level of communities against environmental stress and climate change impacts, it is fundamental to understand peoples’ behaviour. Assessing and enhancing the effectiveness of climate change adaptation will be accomplished by including beneficiaries in the process.

## Research sites

In 2013, expert consultation, site visits, and interviews with local people and stakeholders identified seven study sites. An effort was made in the site selection process to include a diverse set of livelihood systems that were experiencing extreme environmental adversity. The objective was to include the three principal stress clusters affecting the country:

- Cyclones on the coast.
- River bank erosion in flood plains.
- Drought in water-stressed areas in North-Western Bangladesh.

In 2014, visits to the study sites conducted multi-methodological fieldwork that aimed to identify how people were affected by, and adapted to, stressors, with or without support from the government and NGOs.

**Fig. 1:**
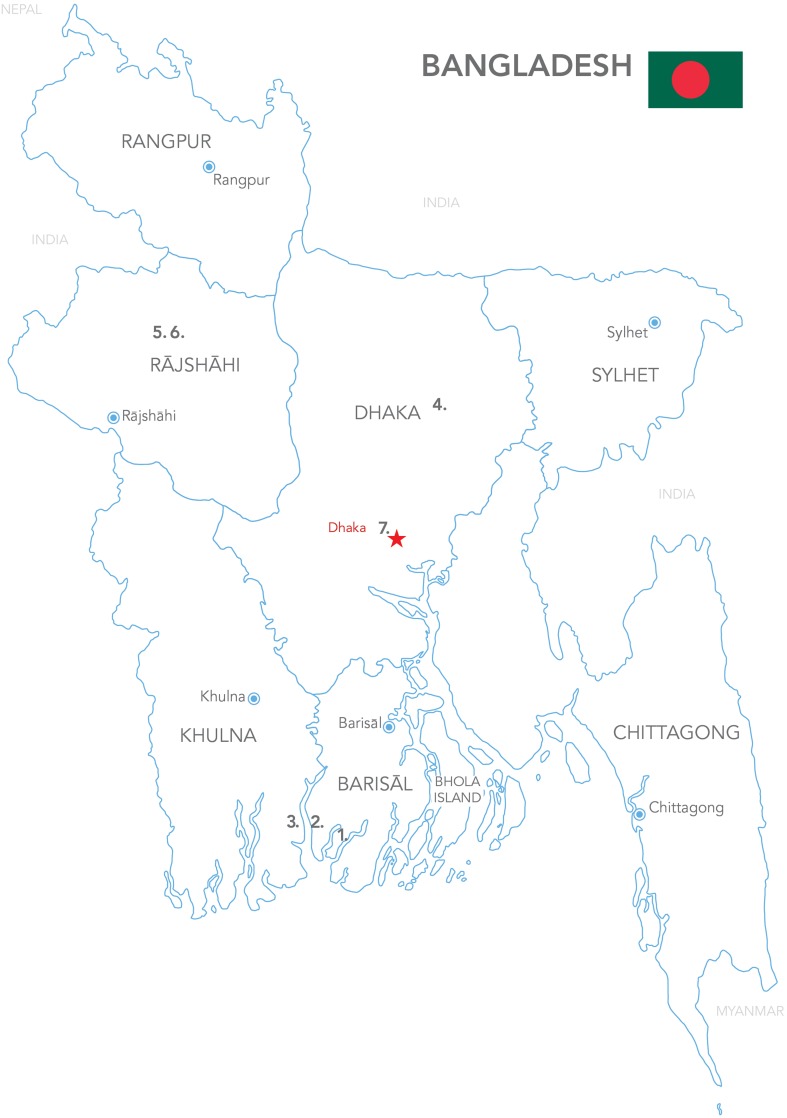

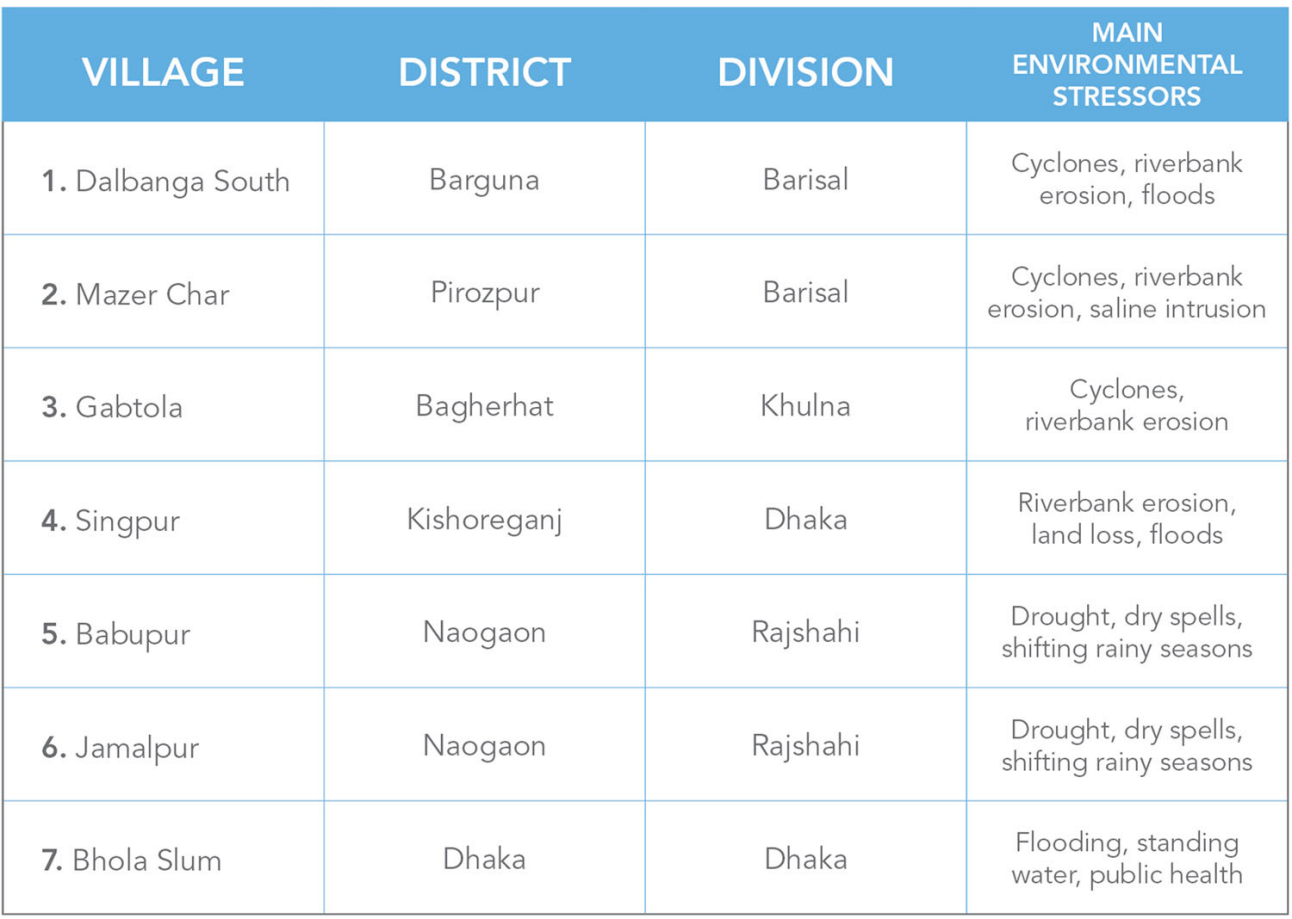
Map showing study site locations and table of division and district locations as well as environmental stressors in the surrounding areas. *Map and Table* prepared by Aileen Orate, UNU-EHS/UNU-VIE Communication Unit 2015

The rationale for choosing those seven study sites in Bangladesh was that places with long historical experience of climate-related stress can provide valuable insights about confronting climatic challenges. These insights are valuable to regional and international communities around the world that may have to deal with related stress. As observed on the map (see Fig. 1), three study sites (*Dalbanga South*, *Mazer Char*, and *Gabtola*) are located in the southern coastal delta, dealing mainly with riverbank erosion, cyclones, and floods (Nadiruzzaman and Wrathall 2015). Two sites (*Babupur* and *Jamalpur*) are situated in the north-western drought affected side of the country. The last two sites (*Singpur* and *Bhola Slum*) are centrally located. The Singpur community is facing riverbank erosion, land loss, and floods, while Bhola Slum, in the capital Dhaka, was added to investigate what happens to people after migrating from rural to urban areas due to environmental stress. The slum is named after Bhola Island, from where most of the inhabitants migrated after cyclones and abrupt riverbank erosion (McNamara et al. 2015).

## Methodology

**Fig. 2:**
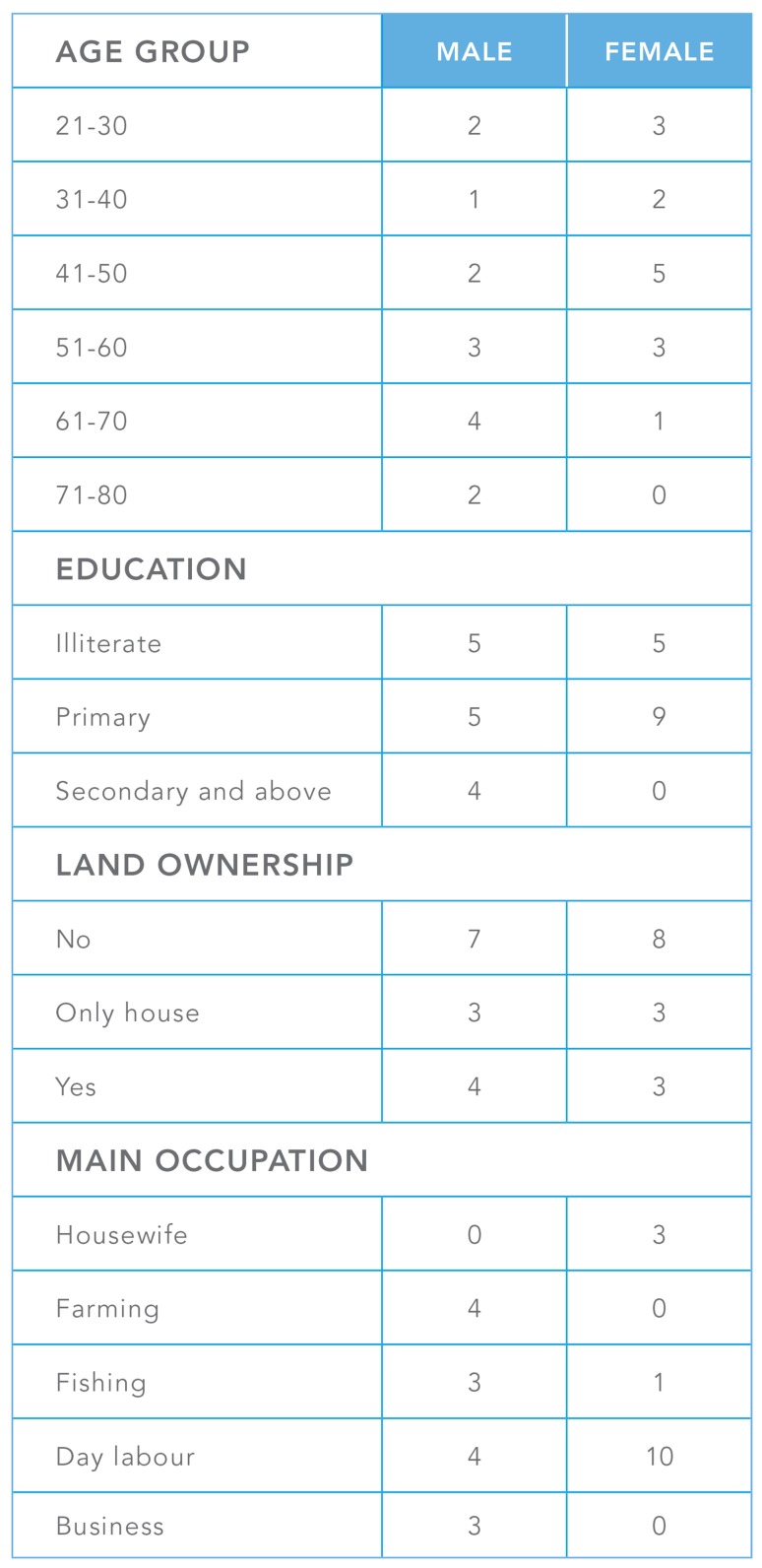
Overview of age, gender, education, landownership, and main livelihood occupation of the Livelihood History informants. *Table* prepared by Aileen Orate, UNU-EHS/UNU-VIE Communication Unit 2015

The fieldwork focused on understanding the diverse impacts of environmental stress on livelihood resilience. In addition, it included a participatory assessment of the effectiveness of the past and present adaptation policy and projects in the study sites (Tschakert et al. 2013; Kumar 2002; Dietz et al. 2013). The qualitative method used in this article, livelihood histories (LH), is individual life histories that focus on changes in people’s livelihoods (Van der Geest 2004). A livelihood perspective in development research and practice enhances understanding of the complexity of rural development (Scoones 1998, 2009; Cannon et al. 2003). The LHs address a common critique of livelihood research: that it tends to be a-historical (De Haan and Zoomers 2005). Reconstructing changes in people’s livelihoods over time, using semi-structured interviews, allows a unique and dynamic view of how environmental stressors and shocks affected people; how they coped with immediate consequences; how they tried to recover; whether they were successful; and what the barriers to full recovery were. Twenty-eight LHs in total, four in each study site were reconstructed. A gender, age, and class balance were maintained in the selection of informants. To ensure that the research findings identified potential gender differences half of the 28 informants were men and half women. The youngest informant was in his 20s and the oldest in her late 70s (see Fig. 2). As the Livelihood History interviews aimed to capture livelihood changes over time, older informants were intentionally oversampled. People involved in livelihood activity for a couple of decades will be able to tell more about changes and stress over a longer period. Communicative informants were overrepresented due to the open narrative structure of the interviews. The field schedule included time for conversation with several community members before starting off the interview sessions. Most informants were identified during other field research sessions, such as the transect walk, PRA sessions, or institutional landscaping. During the sampling, the researchers made sure that the livelihood activities, climatic experiences, and living conditions reflected the study site communities. People who had recently migrated to the community, or had spent a notable amount of time living elsewhere, were not selected, as the adaptation strategies aimed to reflect the history of study sites. The informant sample aimed to reflect the balance of livelihood activities and socio-economic foundation of the communities.

Before the interview, informants were given a general description of the project and the aims and intended use of the research findings was clarified. The interviews took 3–5 h each, and the interview sessions were spread out over 2–3 days to avoid response fatigue. The researchers faced some minor challenges with informants dropping out due to livelihood activities and time constraints. The fact that the interview sessions were spread out over a couple of days helped, including less flexible informants. The researchers used a two‐page checklist containing about 30 topics, but the story and perceptions of each respondent guided the interview. The field team consisted of three international (from Europe/US) researchers and seven Bengali speaking researchers. Translations into Bengali of research topics, concepts, and tools were critically discussed before the research sessions within the field team. During the research sessions, the international researchers worked with at least one Bengali speaking researcher who acted as interpreter.

The informants were given a choice to approve or decline the recording of the interview. A consent form in Bengali was signed allowing the material to be published for non-commercial use. This consent form was read and discussed with the informants before and after the interview sessions. Return visits to clarify uncertainties in the interviews have taken place. All research data were recorded through audio recorders, notebooks, and data entry templates. Transcription, and data entry into electronic documents in English, was scheduled on a daily basis after each set of research sessions.

Qualitative research on people’s responses to environmental stress provides decision and policy makers with crucial insights that support the creation of sustainable climate policies. Oliver-Smith (1996), for example, argues that understanding disasters, stress, and shocks from an anthropological and societal viewpoint are crucial. Disasters, environmental stress, and shocks help signal the response processes and failures of a society to adapt successfully to specific features of its natural and socially constructed environment (Blaikie et al. 2014; Cannon and Müller-Mahn 2010). That is why it is important to understand how and why people’s adaptation strategies sometimes fail, or why people do not adapt at all. Understanding the sustainability of a society is critical to its survival and increased resilience level (Oliver-Smith 1996; Brown and Westaway 2011). The greater cross-disciplinary communication and collaboration between quantitative and qualitative scientists, within the field of disaster research, is a result of the increased urgency surrounding the topic. The study of human–environment interaction is a key to address social issues and problems which contribute to the complexity and difficulties of disaster response, resilience level, and adaptive capacity (Oliver-Smith 1996; Grothmann and Patt 2005; Tompkins and Adger 2004). Adger (2010) argues that policy discussions frame the impacts of climate change as a threat to nation-states, so the discourse has moved away from people, and their capability to adapt to imposed harm and human wellbeing. Human security provides a broader understanding of climate security than one focused on the security of states. That is because it includes justice, adaptation, and vulnerability (Adger 2010).

This journal article presents a qualitative, people-centred, research approach achieved through the LH interviews. The objective is that such an approach will move the focus back to peoples’ adaptation strategies and the constraints they face when confronting environmental stress and shocks. Individual’s conceptual understanding of the causes and meanings of events is crucial within anthropology, philosophy, sociology, behaviour economy, and social psychology (Callahan and Elliott 1996; Patt and Schröter 2008; Patt and Schrag 2003). The LH approach helps present clear insights in how people communicate meaning and understand specific events. The experimental design of ‘reconstructing stories’ is a free narrative approach that ensures a rich data collection that is especially valuable for understanding everyday behaviour.

## Results

The diverse environmental stressors identified in the seven study sites disturb, interrupt, and shock people’s livelihood systems, in particular, those who depend on natural resources.

### Reports on how environmental stress and shocks disturb people’s livelihood resilience

The research in the study sites looked at factors that enhance and undermine resilience. A wide range of adaptation strategies that people use to confront environmental stress, but also the numerous constraints they face, were revealed. Climatic stress puts pressure on vulnerable populations struggling with poverty, food scarcity, land degradation, and lack of adequate housing (Adger et al. 2003; Leighton 2011; Cannon 2000). The environmental stressors typical in the study sites, such as cyclones, floods, riverbank erosion, and drought, can result in livelihood instability (Huq et al. 2013; Warner and van der Geest 2013).

Bangladesh’s location in the largest delta in the world means that an immense number of rivers flow through the country. These rivers provide livelihood options for the people in the study sites, but they simultaneously stress people’s livelihood systems and assets (Ahmed et al. 2012; Lewis 2011). Land loss, through riverbank erosion, is common in study sites, such as Dalbanga South, Mazer Char, Gabtola, and Singpur. These coastal villages move with the erosion as people follow the riverbank. Several respondents report having lost, rebuilt, and again lost, two or sometimes three houses into the river. A number of informants have been forced to sell the little land that remained to enable a shift in livelihoods. For example, selling land for money to purchase fishing nets or a fishing boat is mentioned by several informants in southern study sites. One of the Livelihood History informants, in the study site Dalbanga South, describes how her living conditions took an abrupt change due to the riverbank erosion in the following way:

When the riverbank erosion started we lost happiness in life. When I was a child we used to eat rice from our own fields. After [the riverbank erosion] we never got to eat rice from our fields again./…/There used to be three roads, three villages and three wells by the riverside but they all went under water, into the river. The food scarcity came with the riverbank erosion [Female (1955), Dalbanga South, 2014.05.18].^4^

That is an example of how riverbank erosion permanently reduces natural resources linked to livelihood systems and food security. The informant’s family was farmers for generations and heavily depended on natural resources, such as fertile soil and water. The harvest did not only put food on the table but was also the family’s main source of income. In Singpur, another informant expresses a similar experience of losing’everything’ to the river:

I experienced the extreme effect of riverbank erosion in 2010 when 25 houses [in the village] went under water over a night. Several crop fields were also damaged. We lost everything./…/We never managed to overcome the damages of this disaster. Now we are close to being landless people (Male (1959), Singpur, 2014.06.02).^5^

‘We lost everything’, is one of the most commonly used expressions in the Livelihood History interviews. Clearly, the informants did not lose ‘everything’, but it is a strong way of conveying how severely their living conditions and livelihoods have deteriorated because of the stress. The informant’s statement reveals his incapacity to recover or bounce back from the shock. He lost hits house and a significant amount of land to erosion. He may not have lost everything, but for a farmer, the loss of land feels like losing everything. Loss of livelihood, translates into loss of food security, wellbeing, health, and other essential needs. It constitutes a risk to people’s survival (Cannon et al. 2003; Warner and van der Geest 2013). An informant in Mazer Char recounts his loss during cyclone Sidr (2007):

We were doing quite well until Sidr/…/with 19 cows producing milk that I could sell for 50 taka/litre, and combining it with selling the fish that I managed to catch each day. I was pretty well off economically./…/Then it was that day that changed my life, the day when 10 out of 19 of my cows died, the day I lost my wife [Male (1968), Mazer Char, 2014.05.22].^6^

Sidr was an enormous shock to the informant’s livelihood security. It was such a shock that he specifies the exact number of cows that died and why they were so important to him. Only then, does he mention the loss of his wife. That does not mean that the death of his wife was less important. However, it rather emphasises the importance of losing his livestock; the livestock represents security of livelihood, food, health, family, and life. Since the family could not afford to lose their livestock, they chose not to evacuate to the cyclone shelter. They stayed to protect their livelihood security, and as a result of that, his wife died. The abundant vegetation and forest protection on Mazer Char make the island very suitable for keeping livestock. People let their livestock walk around freely on the island, and they do not need to provide them with food. However, when environmental shocks, such as cyclones and floods, occur, people can lose in a single day the herd that they have accumulated over years (Chowdhury et al. 1993; Alam and Rahman 2014). In Singpur, agricultural land gets flooded every year during the rainy season. Farmers suffer crop losses when the floods arrive earlier than usual. People in Singpur cannot farm during the rainy season because of the annual floods. They pursue alternative livelihoods options, often in other places, which results in yearly seasonal migration.

In the northern study sites, drought and dry spells create difficulties for farmers and put pressure on people’s livelihoods:

Hot waves [drought] can destroy the crops. Due to the hot wave the paddy burns out and ends up without rice in it. When the crops start to have paddy and it does not get water, the paddy turns into patan [rice-less paddy]. Deep tube-well water is expensive. The people who have ponds only use the water for their own land./…/It happened to us 4–5 years ago, half of our paddy turned into patan./…/We had to take loans from the rich people to survive that year [Female (1964), Babupur, 2014.06.07].^7^

Our dry season starts every year in the Bengali month of Chaitra [March–April] and lasts until Jaistha [May–June] but it is also dry during Ashin [Sept–Oct]. This time is the driest period here. We can not get water for irrigation so the rice production goes down [Female (1989), Babupur, 2014.06.05].^8^

In the northern study sites, migration is a common seasonal adaptation strategy in dealing with changes in rainfall patterns, dry spells, and drought:

This year my husband already went to Dhaka twice and he was planning to go now again, but then we got some rain so he will find work here in the village now. That is why he did not go this time./…/My husband goes to Dhaka during drought [and dry spell] because those periods mean less crops and less work [Female (1983), Jamalpur, 2014.06.09].

Seasonal environmental stress, such as the dry season in the north-eastern study sites, also results in disturbances of wellbeing and health. People temporarily lose access to drinking water, forcing them to search for fresh water in neighbouring areas:

Our village’s biggest problem is [access to] drinking water./…/We have to go to other villages to get water./…/The problem is more serious during Magh, Falgun and Choitro [winter/spring] [Male (1982), Jamalpur, 2014.06.09].^9^

In Babupur, deep tube-wells help people to maintain access to drinking water throughout the year. In some areas of the village, however, the groundwater level is low and the tube-wells dry up. While deep tube-wells can be a successful short term adaptation strategy to a lack of rainfall, it is maladaptive in two ways. First, it reduces access to water for those who cannot use the deep tube-well, and second, it reduces access to water in the longer term because of its effect on the groundwater table (Shahid and Hazarika 2010; Raleigh and Kniveton 2012). In Jamalpur access to fresh water is even more limited, and people face conflicts and attacks from neighbouring village members when trying to collect drinking water from their tube-wells. Worldwide studies show that loss of natural resources, and limited access to water and land, for example, can result in violence between people. As natural hazards, stress, and shocks become worse due to climate change impacts, adaption planning needs to take social pressure and disturbance into consideration (Homer-Dixon 1994, 2009; Percival and Homer-Dixon 1998; Raleigh and Kniveton 2012; Raleigh et al. 2015).

Bhola Slum in Dhaka is a common destination for migrants from Bhola Island. Those people usually move there permanently. The Island is becoming uninhabitable due to extreme environmental stress, but not everybody who moves wants to move. Climate change and environmental stress can push people into impossible situations that force them to leave their homes. In such instances, global policy frameworks should be able to protect and support vulnerable people in their decision to move (McNamara et al. 2015; Black et al. 2011a, 2013; Kniveton et al. 2012; Adger et al. 2003). Future adaptation planning, therefore, needs to include a critical assessment on how to support and assist those who consider migration an option. While forced displacement due to climate change and environmental stress needs to be avoided to the extent possible (Black et al. 2011a, b; Adger et al. 2003). The environmental stressors and shocks people face in this study puts pressure on their livelihood systems and wellbeing. Adaptation strategies to such stress can be successful by enhancing resilience. However, our findings also reveal that adaptation measures at times fail and undermine livelihood resilience.

## Adaptation strategies and constraints

This section addresses the questions on how people adapt, absorb, and recover from environmental stress and shocks, and in what way the adaptation strategies enhance people’s livelihood resilience as well as their constraints to do so. Agricultural adaptation, alternative livelihoods, and migration are the most common strategies identified in the study sites.

### Modifying agricultural practices

A large number of people in Bangladesh have agricultural livelihoods that depend on natural resources and are sensitive to climatic disturbances. People struggle to bounce back from extreme weather events and natural hazards when their livelihood systems fail them (Blaikie et al. 2014; Corendea et al. 2012; Kloos et al. 2013). To make their livelihoods less sensitive to a volatile climate, farmers interviewed in the northern study sites have adapted their farming practices to cope with the drought.

In Babupur, farmers described how, using the pond and deep tube-well water for irrigation, they managed to go from one to two or three paddy harvests a year. Another adaptation strategy in the northern study sites was to switch from paddy to cultivation of fruit or vegetables that required less water:

I was the first man who started mango farming in this village/…/I used to do share cropping on Porsha’s [other people’s] land. I was told to try mango farming on my land and so I did./…/I benefited from the mango cultivation as I think it [mango] grows better here in the northern area [Male (1964), Jamalpur, 2014.06.08].^10^

I do not know whether it is good or not but the fact is that nowadays, mango farming is more profitable than paddy cultivation./…/Drought can not harm mango farming that much when the trees are small, they need water, but after a while they can survive without [almost] any water [Male (1987), Babupur, 2014.06.05].^11^

The informants tried to adjust their livelihoods when they perceived an increased risk of a temporary loss of access to water. When viable adaptation options exist, most informants adapt their livelihoods to protect themselves against food and livelihood insecurity. For example, farmers in Babupur and Jamalpur described going from farming rice to farming mango or water melon. That meant they were able to reduce the risk of drought-induced loss or damage to the rice crops. Another way to face the climatic stress in Babupur and Jamalpur has been the construction of ponds. Pond-owners can use the water for their fields during dry spells and drought, sell pond water, or rent out water pumps:

Sometimes I sell my pond water and sometimes I only rent the pump. I do not sell my pond water when I need it myself. The hourly rent for my water pump is 120 taka and the total price for pond water and water pump rental is 400 taka/h [Female (1949), Jamalpur, 2014.06.08].^12^

When people lose access to natural resources, they are forced to seek elsewhere for alternatives. The increased demand for water creates a market. Farmers facing the risk of losing crops will do everything possible to minimize that risk. Ponds are usually used to keep fish and for household or hygiene purposes. Nevertheless, during a dry spell or drought, the function changes and they become a source of income and security. Sharecropping or shared ownership of livestock can be another way of reducing the risk of loss due to environmental stress, but it can also be a highly exploitative system. In Jamalpur, one informant described the system in the following way:

I do not have any land. I have taken two Adi [share] cows and now they have become three. We will sell them and distribute the money [between us] as 50/50 %. I also have three Adi goats. We got three Adi goats [from an NGO] and from them we got three more. I want to sell them but the price is not that good right now. So we are waiting. When the price goes up we will sell them and distribute the money into 50/50 [Female (1983), Jamalpur, 2014.06.09].^13^

The positive side of sharecropping and share-ownership of livestock is that people do not invest as much money when buying livestock or cultivating crops. They divide the offspring or harvest between themselves, and if there is a loss, due to environmental stress, shocks or other adversities, they lose less. When there is a loss, those who are already poor and vulnerable may end up in debt or without food, leaving them even more vulnerable than before the investment. In drought-affected areas, such as Babupur and Jamalpur, or in flood prone areas, such as Singpur, these systems are common. These sharing systems sometimes result in decreasing people livelihood resilience, and sometimes increase their ability to bounce back from natural hazards.

### Switching to alternative livelihoods

An important adaptation strategy to reduce people’s vulnerability to environmental stress is to diversify livelihood sources or shift to livelihood activities that are less dependent on natural resources (Scoones 1998; Cannon et al. 2003; Ellis 1999). After losing land to erosion, one informant in Dalbanga South described switching livelihood from farming to fishing:

I used to cultivate my own land and then I had to switch to share cropping. Lately I have been fishing. I started fishing because our income was not good enough and I do not have the strength anymore to cut mud [do day labour]. If I manage to catch at least 50 fish [a day], then I can buy the essential things I need. If I get 200 then I get something extra for my wife [Male (1944), Dalbanga South, 2014.05.19].^14^

Limited availability or loss of access to natural resources sometimes creates tension between people (Raleigh and Kniveton 2012; Raleigh et al. 2015; Homer-Dixon 2009; Kloos et al. 2013; Matthew 2008). Conflicts have arisen in Dalbanga South around ‘who is allowed to fish where’ and ‘who is allowed to sell fish, and to whom’:

At first the/…/family and the/…/family used to do business together, but then three people came here from Barguna and decided to take up the same business. The newly arrived said; “*You are fishing on our land and you are cleaning out our waters. Are you not going to include us in your business*? *If you want to do business alone we will see how well you will manage to continue the business* [Male (1939), Dalbanga South, italic mine, 2014.05.18].^15^

The family wanting inclusion in the fishing business lost their land to riverbank erosion. That led to a conflict around access and ownership of natural resources, as fish captured by the informant were swimming in water above the family land. Land that is now under water due to the erosion. It is not uncommon for such conflicts to turn into violence. Studies show that conflicts over land and water also become more frequent and severe as access to these natural resources becomes scarcer (Raleigh and Kniveton 2012; Raleigh et al. 2015; Homer-Dixon 1994, 2009). Our study site, Mazer Char, is a relatively new island. Groups of landless people moved there, many of whom occupied land without registered ownership. Land ownership and access to land have been a source of conflict and violence on the island. One informant describes one such incident:

They tied him up with ropes, hands and feet, so he could not move and then they started beating him up. They used wooden sticks and took turns to beat him up until he was unconsciousness. Then they spit paan [betel leaf with areca nut sometimes chewed with tobacco] on him and told him to leave and stop occupying land that did not belong to him./…/After that they threw him in the river. If it was not for a man passing by, finding him half dead in the water and bringing him back home to us. He would have died (Female (1963), Mazer Char, 2014.05.24).^16^

The informant is referring to her father who was beaten up badly, because the family was living on land that was not theirs. As a result, her father lost function in his hand and arm. The conflict was a shock to their livelihood security, since the father was no longer able to work and provide for his family.

Most inhabitants on Mazer Char have two or three sources of livelihood, which makes them more resilient to environmental stress. If one livelihood source is lost or temporarily less productive, due to environmental stress or other disturbances, the inhabitants can rely on the other source. The high tide prevents the crops from growing well and sometimes tidal flood results in crop failure. Hence, people lay out their fishing nets early in the morning, come back to care for their crops and return to collect their nets in the afternoon. Besides farming and fishing, many also keep livestock that allows them to sell milk and meat. Half of the island belongs to the Bangladeshi forest department and people let their livestock walk around free on the land. Fishing is another livelihood source. It strongly depends on a healthy ecosystem. Coastal and river-based fishing are illegal for 3 months a year. It is common for people, whose livelihoods are threatened to fish illegally at night or engage in other illicit activities. The disincentives to illegal livelihood activity are unclear and informants describe gaps in the legal system:

You never know for sure when it comes to fishing./…/When fishing in rivers you sometimes end up with a lot of fish and sometimes with nothing. Fishing is an occupation that if I am lucky allows me to earn up to 500 taka/day, but if I am unlucky as little as 100 taka/day. It is not a very stable profession since the income varies a lot/…/by doing [timber] day labor in the Sundarbans you earn a fixed amount of money [Male (1968), Gabtola, 2014.05.26].^17^

What is illegal? Some military men come here to hunt deer. That is completely prohibited. The police joined them too. They are the people who are supposed to protect the law and they hunt deer. So please tell me what is illegal and not [Female (1967), Gabtola, 2014.05.26].^18^

Gabtola and Mazer Char are located next to the Sundarbans protected area. It is the largest single block of tidal halophytic^19^ mangrove forest in the world and a UNESCO World Heritage site since 1997 (Giri et al. 2007). No trees may be cut down without permission from the national forest department. Illegal logging is considered a serious crime that can result in a prison sentence. The risk people put themselves in, shows the lack of sustainable livelihood options in the area. The risks around such livelihood alternatives may seem extreme, but when losing food security people do what they can to survive:

We go to the Sundarbans not because of greed but to support our stomachs. If we do not go there then what should we do to feed our families? It is not an easy job to cut wood in the Sundarbans, it is hard work [Male (1968), Gabtola, 2014.05.26].

An example of successful livelihood diversification comes from the northern study site of Babupur. A farm labourer was able to save some money and decided to make an investment that made his livelihood more sustainable:

I think it was a good decision to invest in a harvest machine. I worked on other people’s fields before on contract based labour./…/I did not have anything then but now I can earn some money and with that money I can feed my family./…/My sons and I use the machine to separate rice and receive rice as payment [Male (1959), Babupur, 2014.06.04].^20^

Taking advantage of new livelihood opportunities is one way to adapt to limited, reduced or unavailable natural resources (Scoones 1998; Cannon et al. 2003). Adaptation strategies like these are still sometimes not enough, to cope with extreme events and natural hazards. Even though an adaptation measure works well in normal years of stress, it might not sustain in the case of climatic shocks. For example, cyclone Alia in 2009 destroyed the land of several farmers on Mazer Char despite adaptive measures that aimed to protect the land.^21^ Sudden environmental shocks can interrupt or destroy people’s adaptation processes (Adger et al. 2003; Black et al. 2013; Warner and van der Geest 2013). Neighbouring study site Gabtola is facing similar stress and shocks:

When Aila struck we did not really face any severe loss of lives, but the crop fields got completely destroyed due to the saline water. Even today, we still can not really cultivate large amounts of our fields since the land still has not recovered its productivity [Female (1967), Gabtola, 2014.05.27].^22^

When people are unable to cope and adapt locally, temporary, seasonal or permanent migration is another option (Black et al. 2011a; Kniveton et al. 2012; Adger et al. 2003; Oliver-Smith 2009). Migration, in the search for livelihood opportunities elsewhere, can be an escape from a temporary or permanent failure of a livelihood system.

### Migration as an adaptive strategy

People have used migration as an adaptation strategy for centuries; they move away from stress and towards places that help sustain their wellbeing. People use migration to deal with a permanent loss of livelihood options in their home areas. Short-term migration can also help overcome temporary livelihood insecurities, due to climatic stress and other disturbances (Black et al. 2011a, 2013; Kniveton et al. 2012). Migration can be an effective way to bounce back from environmental shocks. When people, for example, already have a social network in place elsewhere, such as Bhola Slum, the adaptation strategy is more likely to be successful. National government and NGOs can in such instances economically support people who consider migration as an adaptive solution. Assisted migration programs have a greater chance of success than inflexible resettlement programs. By contrast, forced displacement can also be an indication that the previous adaptation strategies have failed (Black et al. 2011a, 2013; Adger et al. 2003). Seasonal, temporary and permanent migration is common in all seven study sites:

I went to Sylhet to cut wood in a saw factory. I go there every year for 2–3 months./…/My daughter also went to Dhaka to work in the garment factories. Now she is earning 7000–8000 taka a month [Male (1963), Gabtola, 2014.05.27].^23^

I usually go to Dhaka when there is no work to do here. After [Bengali month] Magh the crops are harvested so there is no work for us. That is why we go to Dhaka in [Bengali month] Falgun and spend the whole month there. After 1 month we return to the village for about 15 days and then we go back to Dhaka. We go to Dhaka to work as day labors [Male (1982), Jamalpur, 2014.06.09].^24^

I went there because we did not have any money left in our family to survive. So I was almost forced to go and work for a living [Female (1984), Singpur, 2014.06.02].^25^

In 1995 we all came to Dhaka. I could not continue living there [Bhola Island] after my parents passed away. We had already lost most of our land to riverbank erosion and our house was located very close to the river. We had no other option than to come here. That is why we moved to Dhaka [Male (1952), Bhola Slum, 2014.06.12].^26^

In the study sites, seasonal migration is common, where ecosystems are seasonally stressed. A good example is the northern study sites during the dry season, or in Singpur, where, during the annual flooding, people cannot farm for several months:

When there is no work on the fields, they [my sons] normally go to Dhaka./…/The money we can earn here is not enough for the year. So to make a decent living we have to go to Dhaka./…/In Dhaka we can earn 500–600 taka/day. We thought of going to Dhaka for the first time when we saw others doing so. Other villagers and people from other villages went to Dhaka to work for a better living so we thought, why are we sitting here [during the dry season] and doing nothing [Female (1949), Jamalpur, 2014.06.08].

I usually go to Dhaka during the rainy season because then we do not have anything to do in our village. If I go to Dhaka to work, it helps my family to survive [Male (1990), Singpur, 2014.06.01].^27^

Seasonal migration can be a successful adaptation strategy that enhances livelihood security. However, it often comes at a cost; working conditions tend to be challenging and dangerous. Migrants are often injured or even killed in work-related accidents (McNamara et al. 2015). Loss of ability to work means income loss and reduced livelihood resilience. When people get sick or injured, they usually need medicine or immediate hospital attendance. Migration can serve as a solution to escape environmental stress, but it also exposes people to new hazards and risks:

My husband can not work properly as he had an accident. While cutting mud on a hill he was struck by a sudden landslide. There was a pipe inside the hill and it broke creating a landslide and he fell down in a hole got buried. The other workers removed the mud and managed to save him. They took my husband to the hospital. Now when trying to work he faces a lot of problems. He has pain coming from two sides of his belly and sometimes when he coughs, blood comes out of his mouth [Female (1975), Bhola Slum, 2014.06.15].^28^

Carrying bricks is not the nicest job. It is hard and it breaks you down. I know that you must think that I am older than 35, but I am not. I am quite young. It is the effect of the hard work [Female (1981), Singpur, 2014.06.01].^29^

Many people from this village went to Alliganj after losing everything [previous crop failure]. They went to Alliganj because anybody can get work there. Your whole family can work there which is impossible here. Though they have to work hard in Alliganj, which makes them forget about their [additional] family back here [Male (1990), Singpur, 2014.06.01].^30^

People who are forced to migrate permanently from rural to urban areas often end up in slums with difficult living conditions. In 1970, Bhola cyclone took half a million lives in Bangladesh. Others lost homes and land and migrated to Dhaka. The migrants built a slum, which they named after their home island: Bhola Slum. Inflow of migrants from Bhola Island continued, due to subsequent cyclones, land loss and riverbank erosion (McNamara et al. 2015):

I came to this place because my father had a lot of property on Bhola Island, but with the riverbank erosion everything went under water./…/When we came here, we made our houses on top of bamboos pillars making sure it was high enough for the water not to enter the house./…/The slum was about 200 hands down when we first came here. Even some trollers and launches [boats] used to depart from here. After that we filled up the water with garbage, to create land where there was water. /…/ Then we had to buy sacks filled of mud at the cost of 10 taka/bag to cover up the garbage and create land [Male (1945), Bhola Slum, 2014.06.12].^31^

Our house was quite far from the river so I never thought that the river would get that close so fast. I never thought the house would go under water so quickly. When also my house went into the water due to the erosion, that is when I came to Dhaka [Female (1963), Bhola Slum, 2014.06.15].^32^

Sometimes, non-material resources are essential to ensure access to material resources (Ayeb-Karlsson et al. 2015). People who migrate to a place where they already have a social network may have better access to livelihood assets, such as land, housing, and jobs. That increases the likelihood of turning migration into a successful adaptation strategy (Black et al. 2011a, 2013; Kniveton et al. 2012; Warner and van der Geest 2013). New migrants from Bhola Island find such a network in Bhola Slum.

While most informants in the slum indicated that they now earn more money than before migrating, they consider their living conditions to be worse. Migration to the slum involved a substantial loss in health, education, and food security. While the migration reduced exposure to some environmental stress, it exposes them to other risk and hazards. In that sense, the adaptation strategy has failed (Black et al. 2011a, 2013; Bohle 2007; Gonzalez-Parra and Simon 2008):

We now struggle with hunger./…/We have to leave our pride behind and beg for help from others. If I had stayed on Bhola Island, if I still would have my land and house there, I would be all right today. Our living condition would be better and our children would have ended up being highly educated [Female (1975), Bhola Slum, 2014.06.15].^33^

## Policy relevance and future adaptation planning

Any policy framework that aims to address climatic and other environmental changes and shocks as well as to protect wellbeing in vulnerable delta regions must include elements of adaptation, disaster risk reduction, and post-disaster relief (Adger et al. 2003; Folke et al. 2004). Currently, disaster preparedness is often good on paper but not very successful in real life. That can be because of a lack of assessment of community needs, or because of exclusion of the community in the decision-making processes (Keeley and Scoones 2014; Chowdhury et al. 1993; Blaikie et al. 2014). Mistakes are, therefore, repeated. More and regularly repeated community evaluations of disaster preparedness training and early warning systems can help improve the community preparedness. In the longer term, such assessments would help increase people’s livelihood resilience level. For example, if people would have time to put their livestock, fishing nets, and boats in safety prior a cyclone, fewer livelihood assets would be lost. Furthermore, ensuring enough time for people to save their livelihood assets would result in them being able to pick up their livelihoods sooner after a cyclone strike. After a cyclone, organizations should, therefore, try to find out how well the early warning system worked and what improvements are needed (Chowdhury et al. 1993; Alam and Rahman 2014). Serious gaps in the early warning system against cyclones were highlighted in the interviews, as well as the lack of EWS evaluation:

The cyclone EWS is currently not fully functional since the responsible NGO/…/left the community about a year ago and stopped their disaster preparedness training program with the people in the village [EWS FGD, Dalbanga South, 2014.10.15].

I heard about the cyclone at 2 pm but when trying to prepare everything it got too late. We tried getting to the cyclone shelter in the evening but we failed. The roads were already blocked by then with broken trees [Male (1936), Mazer Char, 2014.05.24].^34^

The water flooded in so suddenly, people tried to escape to the cyclone shelter but not everybody managed to reach it. Me and my family, my husband and our three children got caught in the flood and were pulled away. The strong wave took us all the way to the school building where we managed to crawl up on the rooftop [Female (1974), Mazer Char, 2014.05.19].^35^

Policy and adaptation planning, using lessons learned provided by people facing environmental stress, is the first step towards a successful climate change preparedness plan (Grothmann and Patt 2005; Blaikie et al. 2014; Keeley and Scoones 2014; Roy 2009; Setiadi et al. 2010). It is necessary to include the local communities in the decision-making process. A sustainable adaptation plan to climate change must include local knowledge (Van Aalst et al. 2008; Lewis et al. 2003; Reid et al. 2009 Hrelja et al. 2012; Jabareen 2013). Planned adaptation interventions aimed at protecting a vulnerable community, sometimes end up displacing people instead:

If an embankment against the riverbank erosion will be built, it will be on my land because my land and house is on the riverbank. So if the embankment is built on my land then where should I go? [Female (1939), Mazer Char, 2014.05.25].^36^

It is, therefore, crucial to involve people at an early stage in adaptation planning. People-centred research, such as this study, can provide policy makers with valuable local knowledge on social and environmental vulnerability (Keeley and Scoones 2014; Gardner and Lewis 1996).

## Discussion

This article provides a people-centred perspective on environmental stress, natural hazards adaptation, and livelihood resilience. The findings represent a valuable resource for understanding how people can and cannot adapt to environmental stress and shocks in Bangladesh. More people-centred research on adaptation is needed to understand fully the decision-making of societies facing climatic stress. Such studies provide crucial insights that can support people’s future adaptive capacity, and help identify vulnerabilities (Grothmann and Patt 2005).

To summarize the findings, environmental stress, shocks, and disturbances influence people’s livelihood resilience in several ways, and while people adopt a wide range of measures to adapt and recover, these are often not successful. The personal Livelihood Histories showed that people lose livestock and land in the cyclones. They, therefore, sometimes choose not to evacuate, stay behind to protect their livelihoods, and end up putting their lives at risk. Floods and riverbank erosion damage agricultural lands and crops, which may impose food insecurity to people depending on them. However, people can adapt and recover from such environmental stress by modifying their agricultural practices, switching to alternative livelihoods, or using migration as an adaptive strategy. These adaptation strategies strengthen people’s livelihood resilience. For example, when people switch to drought-resistant crops or diversify their livelihoods to include fishing and farming, their livelihoods are more likely to sustain them through a period of stress. People also migrate away from cyclone and flood affected areas to safe places. Nevertheless, the study also finds that sometimes the adaptation strategies fail. Migration can serve as a successful adaptation strategy, but it may also leave people more vulnerable due to poor or dangerous living and/or working conditions. Future policy and adaptation planning must use lessons learned provided by people facing environmental stress. For example, this study highlighted significant gaps in the disaster preparedness and early warning functionality in the coastal study sites. If people had time to move their livestock, fishing nets and boats to safety prior a cyclone, fewer lives, and livelihood assets would be lost. Furthermore, that would result in them being able to pick up their livelihoods sooner after a cyclone strike.

The study confirms the sustainable rural livelihoods framework. People increase their livelihood resilience by adding on an alternative livelihood activity, as this reduces the risk of losing all income sources (Scoones 1998, Scoones 2009). The alternative livelihood activities were often less dependent on natural resources, such as brick and garment factory work. The informants also comprehensively described the inter-linkage between loss of resources, tension in social interactions, and conflicts. Disasters and climate change impacts need to be perceived as stressors that overload the coping capacity of human societies and the resilience level of social systems (Blaikie et al. 2014; Homer-Dixon 2009; Raleigh and Kniveton 2012). People-centred research can help structure people’s responses and behaviour around stress, scarcity, and power relations. As environmental stress and shocks become more frequent and intense worldwide, due to climate change, understanding how people can and cannot adapt provides valuable lessons. People all over the world will, for example, continue to use migration from rural to urban areas as an adaptation strategy. It is, therefore, necessary to better understand not just the number of people migrating, but also people’s decisions, and behaviour in the migration process. Adaptation strategies sometimes fail or add on other risks and pressures to peoples’ wellbeing, health, and livelihood resilience. A more people-centred research approach can help mitigate failures and risks (Black et al. 2011a, 2013; Kniveton et al. 2012; McNamara et al. 2015).

## Conclusion

Bangladesh is in the middle of the world’s largest delta. The delta-based ecosystem nourishes a dense population, but it comes with a wide variety of climatic and environmental stresses, such as cyclones, floods, saline intrusion, waterlogging, sea-level rise, land loss, and riverbank erosion. Droughts and landslides are additional problems that occasionally cause havoc in parts of the country. Among the most vulnerable people in the world are the ones depending on sensitive ecosystems and their providing services. Adaptation strategies, such as agricultural change, livelihood diversification, and migration, can be ways to enhance livelihood resilience. This article argues for the enhancement of global policy frameworks for protecting vulnerable populations against future climate change impacts using a people-centred approach that analyses local experiences with adaptation strategies and constraints. Lessons have already been learned and continue to be learned every day. People-centred case studies, such as the Gibika project in Bangladesh, provide valuable research examples on what people need to increase their livelihood resilience. Furthermore, such case studies help enhance understanding of why people’s adaptation strategies sometimes work and sometimes fail, where the gaps are and how to address them. Future research should include a more people-centred approach focused on the effectiveness of autonomous and planned adaptation measures.

## Footnotes

1 “The capacity of all people across generations to sustain and improve their livelihood opportunities and well-being despite environmental, economical, social and political disturbance (Tanner et al. 2015: 23–24)”.

2 “Adaptation involves changes in social-ecological systems in response to actual and expected impacts of climate change in the context of interacting non-climatic changes. Adaptation strategies and actions can range from short-term coping to longer-term, deeper trans-formations, aim to meet more than climate change goals alone, and may or may not succeed in moderating harm or exploiting beneficial opportunities (Moser and Ekstrom 2010: 1)”.

3 “A process/event involving the combination of a potentially destructive agent(s) from the natural and/or technological environment and a population in a socially and technologically produced condition of vulnerability (Oliver-Smith 1996)”.

4 Livelihood History Interview: Female (1955), Gibika field data, Dalbanga South, 2014.05.18: 12.45–14.25/2014.05.19: 12.00–14.05.

5 Livelihood History Interview: Male (1959), Gibika field data, Singpur, 2014.06.02: 11.34–13.14.

6 Livelihood History Interview: Male (1968), Gibika field data, Mazer Char, 2014.05.22: 14.15–15.59 2014.05.24: 14.45–15.05.

7 Livelihood History Interview: Female (1964), Gibika field data, Babupur, 2014.06.07: 11.20–12.46.

8 Livelihood History Interview: Female (1989), Gibika field data, Babupur, 2014.06.05: 12.11–14.24.

9 Livelihood History Interview: Male (1982), Gibika field data, Jamalpur, 2014.06.09: 11.57–13.20.

10 Livelihood History Interview: Male (1964), Gibika field data, Jamalpur, 2014.06.08: 14.40–16.30.

11 Livelihood History Interview: Male (1987), Gibika field data, Babupur, 2014.06.05: 10.50–12.53.

12 Livelihood History Interview: Female (1949), Gibika field data, Jamalpur, 2014.06.08: 14.38–19.00.

13 Livelihood History Interview: Female (1983), Gibika field data, Jamalpur, 2014.06.08: 11.15–13.23.

14 Livelihood History Interview: Male (1944), Gibika field data, Dalbanga South, 2014.05.19:15.36–17.18.

15 Livelihood History Interview: Male (1939), Gibika field data, Dalbanga South, 2014.05.18: 15.58–17.18/2014.05.19: 11.16–12.31.

16 Livelihood History Interview: Female (1963), Gibika field data, Mazer Char, 2014.05.24: 09.40–12.01/2014.05.25: 14.37–15.03.

17 Livelihood History Interview: Male (1968), Gibika field data, Gabtola, 2014.05.26: 09.47–12.03.

18 Livelihood History Interview: Female (1967), Gibika field data, Gabtola, 2014.05.26: 09.51–11.15.

19 Definition ‘halophyte’: a plant adapted to living in a saline environment (http://thefreedictionary.com).

20 Livelihood History Interview: Male (1959), Gibika field data, Babupur, 2014.06.04: 15.27–18.10.

21 For a similar example from Bangladesh, see Rabbani et al (2013).

22 Livelihood History Interview: Female (1967), Gibika field data, Gabtola, 2014.05.27: 10.30–13.06.

23 Livelihood History Interview: Male (1963), Gibika field data, Gabtola, 2014.05.27: 15.41–17.28.

24 Livelihood History Interview: Male (1982), Gibika field data, Jamalpur, 2014.06.09: 11.57–13.20.

25 Livelihood History Interview: Female (1984), Gibika field data, Singpur, 2014.06.02: 13.30–14.59.

26 Livelihood History Interview: Male (1952), Gibika field data, Bhola Slum, 2014.06.12: 14.40–16.55.

27 Livelihood History Interview: Male (1990), Gibika field data, Singpur, 2014.06.01: 12.09–13.44.

28 Livelihood History Interview: Female (1975), Gibika field data, Bhola Slum, 2014.06.15: 12.27–16.35.

29 Livelihood History Interview: Female (1981), Gibika field data, Singpur, 2014.06.01: 11.59–14.30.

30 Livelihood History Interview: Male (1990), Gibika field data, Singpur, 2014.06.01: 12.09–13.44.

31 Livelihood History Interview: Male (1945), Gibika field data, Bhola Slum, 2014.06.12: 14.40–17.44.

32 Livelihood History Interview: Female (1963), Gibika field data, Bhola Slum, 2014.06.15: 12.32–14.42.

33 Livelihood History Interview: Female (1975), Gibika field data, Bhola Slum, 2014.06.15: 12.27–16.35.

34 Livelihood History Interview: Male (1936), Gibika field data, Mazer Char, 2014.05.24: 15.58–17.18.

35 Livelihood History Interview: Female (1974), Gibika field data, Dalbanga South, 2014.05.19: 15.50–18.10/2014.05.20: 13.45–15.05.

36 Livelihood History Interview: Female (1939), Gibika field data, Mazer Char, 2014.05.25: 09.35–11.45.
